# A 5-Year intervention study on elimination of urogenital schistosomiasis in Zanzibar: Parasitological results of annual cross-sectional surveys

**DOI:** 10.1371/journal.pntd.0007268

**Published:** 2019-05-06

**Authors:** Stefanie Knopp, Shaali M. Ame, Bobbie Person, Jan Hattendorf, Muriel Rabone, Saleh Juma, Juma Muhsin, Iddi Simba Khamis, Elizabeth Hollenberg, Khalfan A. Mohammed, Fatma Kabole, Said M. Ali, David Rollinson

**Affiliations:** 1 Swiss Tropical and Public Health Institute, Basel, Switzerland; 2 University of Basel, Basel, Switzerland; 3 Department of Life Sciences, Natural History Museum, London, United Kingdom; 4 Public Health Laboratory—Ivo de Carneri, Pemba, United Republic of Tanzania; 5 Schistosomiasis Consortium for Operational Research and Evaluation, Athens, Georgia, United States of America; 6 Neglected Diseases Program, Ministry of Health, Zanzibar, United Republic of Tanzania; 7 Schistosomiasis Control Initiative, Imperial College, London, United Kingdom; RTI International, UNITED STATES

## Abstract

**Background:**

The Zanzibar Elimination of Schistosomiasis Transmission (ZEST) project aimed to eliminate urogenital schistosomiasis as a public health problem from Pemba and to interrupt *Schistosoma haematobium* transmission from Unguja in 5 years.

**Methodology:**

A repeated cross-sectional cluster-randomized trial was implemented from 2011/12 till 2017. On each island, 45 shehias were randomly assigned to receive one of three interventions: biannual mass drug administration (MDA) with praziquantel alone, or in combination with snail control or behavior change measures. In cross-sectional surveys, a single urine sample was collected from ~9,000 students aged 9- to 12-years and from ~4,500 adults aged 20- to 55-years annually, and from ~9,000 1^st^ year students at baseline and the final survey. Each sample was examined for *S*. *haematobium* eggs by a single urine filtration. Prevalence and infection intensity were determined. Odds of infection were compared between the intervention arms.

**Principal findings:**

Prevalence was reduced from 6.1% (95% confidence interval (CI): 4.5%-7.6%) to 1.7% (95% CI: 1.2%-2.2%) in 9- to 12-year old students, from 3.9% (95% CI: 2.8%-5.0%) to 1.5% (95% CI: 1.0%-2.0%) in adults, and from 8.8% (95% CI: 6.5%-11.2%) to 2.6% (95% CI: 1.7%-3.5%) in 1^st^ year students from 2011/12 to 2017. In 2017, heavy infection intensities occurred in 0.4% of 9- to 12-year old students, 0.1% of adults, and 0.8% of 1^st^ year students. Considering 1^st^ year students in 2017, 13/45 schools in Pemba and 4/45 schools in Unguja had heavy infection intensities >1%. There was no significant difference in prevalence between the intervention arms in any study group and year.

**Conclusions/Significance:**

Urogenital schistosomiasis was eliminated as public health problem from most sites in Pemba and Unguja. Prevalence was significantly reduced, but transmission was not interrupted. Continued interventions that are adaptive and tailored to the micro-epidemiology of *S*. *haematobium* in Zanzibar are needed to sustain and advance the gains made by ZEST.

## Author summary

Schistosomiasis is a disease caused by parasitic blood flukes of the genus *Schistosoma*. The highest burden is concentrated in sub-Saharan Africa. On the Zanzibar islands, urogenital schistosomiasis has been successfully controlled over the past decades. The Zanzibar Elimination of Schistosomiasis Transmission (ZEST) project implemented from 2011/12 through 2017 aimed to eliminate urogenital schistosomiasis as a public health problem from Pemba and to interrupt *S*. *haematobium* transmission from Unguja in 5 years. In a cluster-randomized trial, we investigated the impact of biannual treatment of the population with praziquantel alone or combined with snail control or behavior change interventions. After five years of interventions, the overall *S*. *haematobium* prevalence was reduced to <3% in schoolchildren and adults. Heavy infection intensities were observed in <1% of the surveyed population groups. Urogenital schistosomiasis was eliminated as a public health problem from most of the study sites on Pemba and Unguja. The prevalence was significantly lower in 2017 compared with 2011/12, but transmission was not interrupted. To sustain and advance the gains made by ZEST, continued interventions that are adaptive and tailored to the micro-epidemiology of *S*. *haematobium* in Zanzibar are needed.

## Introduction

The transmission of parasitic blood flukes of the genus *Schistosoma* is reported from 78 countries. The global burden caused by the disease schistosomiasis was conservatively estimated at 1.43 million disability-adjusted life years in 2017 [1]. The highest burden of disease is concentrated in sub-Saharan Africa [2]. In 2012, the World Health Assembly urged countries to intensify schistosomiasis control and initiate elimination campaigns where appropriate [3].

The Zanzibar archipelago of the United Republic of Tanzania, with the main islands Pemba and Unguja, has a history of schistosomiasis control that dates back to the 1980s [4–7]. At that time, urogenital schistosomiasis caused by *S*. *haematobium*, was highly prevalent across both islands [7–9]. Thirty years later, considerable progress had been made and the overall prevalence in children and adults had decreased to 8% in Unguja and 15% in Pemba in 2011 [10]. Subsequently, the Zanzibar islands were one of the first areas in sub-Saharan Africa where elimination of urogenital schistosomiasis was considered as a feasible goal [10–12].

The Zanzibar Elimination of Schistosomiasis Transmission (ZEST) alliance, consisting of various stakeholders and institutions, including the Neglected Diseases Program of the Zanzibar Ministry of Health, the Public Health Laboratory-Ivo de Carneri, the Schistosomiasis Consortium for Operational Research and Evaluation (SCORE), the Schistosomiasis Control Initiative (SCI), the World Health Organization (WHO), the Natural History Museum London, and the Swiss Tropical and Public Health Institute, aimed to eliminate schistosomiasis as a public health problem from Pemba (<1% heavy infection intensities in all sentinel sites) and to interrupt transmission on Unguja (zero incidence in all sentinel sites) in 5 years [10, 13]. Moreover, it aimed to learn about the effectiveness of snail control and behavioral change interventions. In that regard, the Zanzibar Neglected Diseases Program supported by SCI and WHO, implemented biannual mass drug administration (MDA) with praziquantel to the whole eligible population from 2012 onward. In addition, SCORE funded a 5-year repeated cross-sectional cluster-randomized trial (CRT), which was implemented in Zanzibar from November 2011 till May 2017. A total of 90 shehias (small administrative areas) were randomly assigned to receive one of three interventions: biannual mass drug administration (MDA) with praziquantel alone (arm 1) or in combination with snail control (arm 2) or behavior change measures (arm 3). The primary outcome of the CRT was defined as *S*. *haematobium* infection prevalence and intensity in 9- to 12-year old children after five years of follow-up in 2017; these results are published elsewhere [14]. Here we present the impact of the interventions on the *S*. *haematobium* prevalence and intensity in 1^st^ year students and adults, in addition to 9- to 12-year old children, across all 5 years of the project in Pemba and Unguja, and discuss the implications and challenges for interruption of urogenital schistosomiasis transmission in Zanzibar.

## Methods

### Study site

The Zanzibar islands (Unguja and Pemba) are part of the United Republic of Tanzania. The islands are divided into districts and the smallest administrative areas are called shehias. The population of Zanzibar was estimated at 1.4 million in 2016 [15]. The average population of a shehia is ~4,700 inhabitants but they vary considerably in size (range: 482–26,275 inhabitants) [16]. Most shehias have at least one public primary school. Children usually enter the first grade at the age of 6 or 7 years. Primary schools include grades 1 to 6. The net enrolment ratio in primary schools was estimated at 84.2% in Zanzibar in 2014/15 [15]. The predominant religion in Zanzibar is Islam. In addition to primary school, many children visit religious schools, called madrassas [17]. Among all households in Zanzibar, 90.5% used a protected water source for drinking in the dry season and 83.7% had a toilet facility in 2014/15 [15].

On both islands, urogenital schistosomiasis was considered as a major public health problem in the past century [5, 8, 9]. Repeated MDAs with praziquantel and likely also improvements in the socio-economic standard, including access to clean water and sanitation, contributed to the reduction of the *S*. *haematobium* prevalence. The baseline survey of the CRT in 2011/12 revealed that 8.9% and 4.3% of schoolchildren and 5.5% and 2.7% of adults in Pemba and Unguja, respectively, were infected with *S*. *haematobium* according to a single urine filtration result [18].

### Ethics

Ethical approvals were obtained from the Zanzibar Medical Research Ethics Committee in Zanzibar, United Republic of Tanzania (ZAMREC, reference no. ZAMREC 0003/Sept/011), the “Ethikkommission beider Basel” (EKBB) in Basel, Switzerland (reference no. 236/11) and the Institutional Review Board of the University of Georgia in Athens, Georgia, United States of America (project no. 2012-10138-0). All individuals participating in the parasitological surveys were informed in lay terms about the aims and procedures of the study. Written informed consent was obtained from the parents or guardians of participating children and directly from participating adults. The study is registered with the International Standard Randomized Controlled Trial Number register (ISRCTN48837681).

### Study design and participants

**Fig 1:**
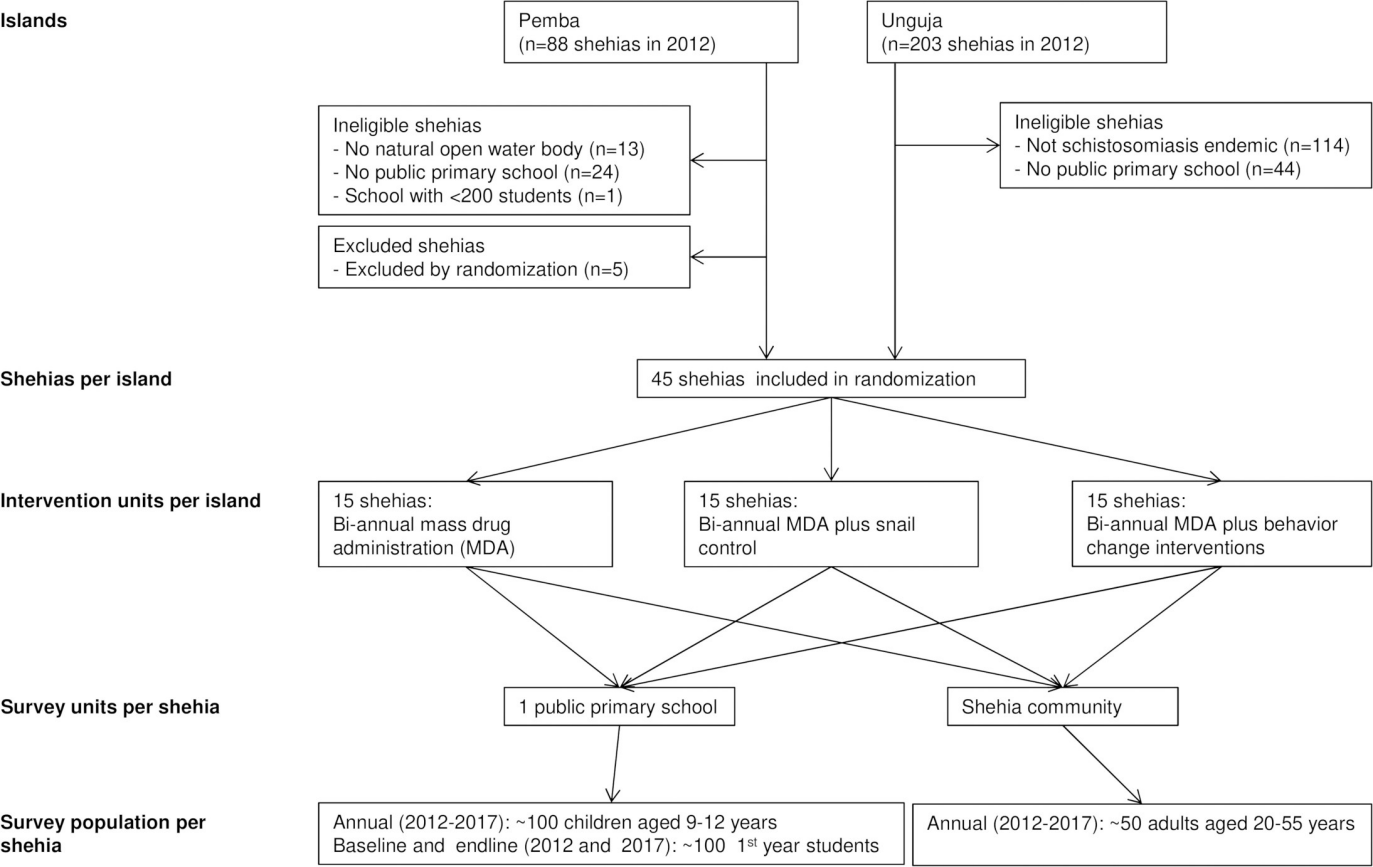
Flowchart of study design.

The study was designed as a 5-year repeated cross-sectional CRT. A shehia was defined as cluster and intervention unit (Fig 1). A total of 45 shehias on Pemba and 45 shehias on Unguja, respectively, were assigned randomly to receive one of three interventions (ratio 1:1:1): biannual MDA with praziquantel alone (arm 1), or in combination with snail control (arm 2), or in combination with behavior change measures (arm 3). In the annual cross-sectional surveys conducted from November 2011 through May 2017, we annually enrolled schoolchildren aged 9- to 12-years from grade 3 and 4, and at baseline and the final survey also from grade 1 in the public primary schools of the study shehias, and annually adults aged 20- to 55-years in the communities of the study shehias. From each participant a single urine sample was collected between 10:00 and 14:00 hours and examined for *S*. *haematobium* infection as described below. In the surveys conducted in 2014 till 2016, 9- to 12-year old students were asked about their participation in the last school-based treatment (SBT) round and adult participants were interviewed about their participation at the last community-wide treatment (CWT) round preceding the survey and about their compliance with praziquantel intake. In all years, the surveys were followed by MDA, where praziquantel (40 mg/kg) was offered to the whole eligible population as described below.

### Randomization and masking

The sample size calculation, eligibility criteria, and randomization procedures of clusters and study participants were described in detail in our published study protocol [10]. Due to the nature of the interventions, neither participants nor field or laboratory personnel were blinded to the intervention arms.

### Interventions

Praziquantel (40 mg/kg) was administered biannually in intervals of approximately 6 months to the whole eligible population by the Neglected Diseases Program of the Zanzibar Ministry of Health [10]. Between April 2012 and November 2016, a total of 10 CWT rounds were conducted. In each round, over a period of 3 days, praziquantel was provided by trained community drug distributors to adults with exception of severely ill people and pregnant women, and to children (≥3 years) who had not received praziquantel during SBT. From 2013, to increase coverage, schools were used as additional venues to distribute praziquantel to children. Teachers, supervised by staff of the Neglected Diseases Program, administered the tablets to students using a dose pole [19]. Treatment in schools was directly observed. Between November 2013 and November 2016, 6 rounds of SBT were conducted in Pemba and 5 rounds of SBT in Unguja.

Snail control was conducted in 15 shehias in Pemba and Unguja, respectively, in addition to biannual MDA. For this purpose, human water contact sites at streams or ponds inside the study shehia boundaries were identified with the support of the local communities. Each site was surveyed up to five times per year for intermediate host snails of the genus *Bulinus* in the dry season, when water conditions were suitable for snail collection [10]. At each visit, the number of *Bulinus* collected by a pre-specified number of fieldworkers in a certain time and length of shoreline was recorded. When *Bulinus* were present, the trained fieldworkers sprayed the molluscicide Bayluscide (niclosamide) to the shorelines near the human water contact site, using Hudson sprayers or a petrol power sprayer.

Behavior change measures in addition to biannual MDA were implemented in 15 shehias in Unguja and Pemba, respectively. The interventions were developed with formative research and using a human centered design approach in 2011 [20]. They were implemented in a phase-in approach and improved over the study period [14]. From 2012, educational play and interactive learning methods for urogenital schistosomiasis prevention were introduced and applied by trained teachers in the public primary schools of the study shehias. Moreover, in each behavioral shehia, one male and one female urinal were installed close to a known transmission site at a river or pond. From 2013, regular behavior change education meetings were conducted in the community. From 2014, to increase the reach to children not attending public schools and to the community as a whole, trained teachers of religious schools (madrassas) were involved as behavioral change agents [17]. In 2014 and 2015, 21 and 25 washing platforms were installed near safe water sources such as taps or wells in Pemba and Unguja, respectively [14]. At least one washing platform was installed in each shehia of the behavioral arm.

### Procedures

The parasitological surveys were implemented annually, starting with the baseline surveys of adults in November and December 2011 and of schoolchildren in January till April 2012. Annual follow-up surveys were conducted between January and May from 2013 through 2017. Each year before the parasitological surveys started, a meeting was conducted with teachers and shehas of all 90 study schools and shehias. The study aims and procedures were explained and the results from the previous years presented. The date when the survey teams would visit the schools and shehias was communicated in a letter to the headmasters and shehas, respectively. Each school was visited on two subsequent working days. On the first day, children were randomized for participation [10]. From enrolled children, name, sex, age, place of birth and their participation at the previous SBT round were recorded. The study was explained to the children in lay terms and they received an information- and a consent-sheet for their parents to sign. On the second day, each child that submitted written informed consent from a parent or legal guardian received a container for urine collection between 10:00 and 12:00 hours. In each of the 90 study schools, urine samples were collected annually from ~100 children aged 9-to-12 years attending grades 3 and 4. At baseline and the final survey, urine samples were additionally collected from ~100 children from grade 1.

Each of the 90 study shehias was visited on one day, for data and urine collection from ~50 adults living in the shehia. The randomization procedures for selection of households and adult participants are described elsewhere in detail [10]. Before enrolment, the study aims were explained to each potential participant by a trained fieldworker and written informed consent for participation was collected. Each enrolled adult was asked to submit a urine sample between 10:00 and 14:00 hours and interviewed with a pretested questionnaire concerning his/her demographic data and participation during the last round of MDA.

After collection, the urine samples were placed in carrier boxes, transferred to the Public Health Laboratory-Ivo de Carneri in Pemba or the laboratory of the Neglected Diseases Program in Unguja and examined during the afternoon of the same day and within seven hours after production. Each sample was examined by eye for macrohematuria and with reagent strips (Haemastix; Siemens Healthcare Diagnostics GmbH) for microhematuria. From samples of sufficient volume, a single 10 ml urine filtration slide was prepared, using a polycarbonate filter with a pore size of 20 μm. The filters were covered with hydrophilic cellophane and a drop of Lugol’s iodine was added to stain *S*. *haematobium* eggs. The filters were examined under the microscope by trained technicians, who recorded the number of eggs detected on each slide. Ten percent of the slides of each technician were kept for quality control by an external microscopist [21]. Once the surveys were concluded, the next round of MDA commenced, where praziquantel (40 mg/kg) was offered to the whole eligible population using a praziquantel dose pole [19].

### Outcomes

The primary outcomes were *S*. *haematobium* infection status and infection intensity among 9- to 12-year old children in 2017 at individual and cluster level. These results are presented elsewhere [14]. The secondary analyses presented here included *S*. *haematobium* infection status and intensity among 9- to 12-year old children and 20- to 55-year old adults in all study years (2011/12 through 2017), and among 1^st^ year students at baseline and the final survey (2012 and 2017). They also included coverage and compliance of the study participants with praziquantel treatment provided in the MDA round preceding the respective survey.

### Statistical analyses

Data were entered in spreadsheets (Excel 2010, Microsoft), cleaned, and analyzed with StataIC 14 (StataCorp.; College Station, Texas, United States of America) and R version 3.4.3 (www.r-project.org). Maps were created with QGIS version 2.14.21 using coordinates of schools collected with a handheld Garmin GPSMAP 62sc device (Garmin, Kansas City, USA) and shape files of shehias provided by the Zanzibar Health Management Information System to the Neglected Diseases Program of the Zanzibar Ministry of Health.

All participants with a urine filtration and/or reagent strip result were included in the analyses. Participants were considered *S*. *haematobium*-positive if at least one *S*. *haematobium* egg per 10 ml urine was detected by the urine filtration method. In the absence of a urine filtration result, a participant was considered *S*. *haematobium*-positive, if microhaematuria was detected with reagent strips. Egg counts were truncated at 1000 eggs per 10 ml urine, and stratified into light (1–49 eggs per 10 ml urine) and heavy (≥50 eggs per 10 ml urine) infections according to WHO guidelines [22]. Based on the manufacturers color code, microhaematuria intensity was graded into negative, trace, +, ++, and +++.

Generalized estimating equation (GEE) models with binary and negative binomial distributed outcomes and independent correlation structure were applied to compare trial arms. Arm 1 was the designated reference group. For unadjusted estimates, trial arm as predictor and school or shehia as cluster, were included in the model. Adjusted odds ratios were calculated using the cluster prevalence at baseline as a continuous predictor in the model. For the inversed probability weighted odds ratios inverse probability weights were calculated for baseline prevalence and number of observations per cluster. The product of both weights was included in the model. All models used GEE with robust standard errors to account for clustering. For better comparability only clusters with baseline information were included.

Coverage in the MDA round preceding the survey and compliance with praziquantel treatment was assessed in 2014, 2015 and 2016. Coverage of schoolchildren was calculated as the proportion of children, who participated in the directly observed SBT, among all 9- to 12-year old schoolchildren surveyed. Compliance of adults was calculated as the proportion of adults, who reported in the questionnaire interview to have had received and swallowed praziquantel tablets all together (instead of not taking the tablets at all, or splitting intake to morning and evening, or over multiple days), among all surveyed adults [23].

## Results

### Study flow and baseline characteristics

Pemba consisted of 88 shehias and Unguja of 203 shehias in 2011. Among them, 201 shehias were excluded in a stepwise procedure and finally 45 shehias on Unguja and Pemba, respectively, were randomized to one of the three intervention arms as shown in Fig 1 and described in detail in the published study protocol [10]. The number of children and adults in each study arm included and excluded from the analysis of annual cross-sectional surveys is indicated in S1 Table. The baseline prevalence of *S*. *haematobium* in arm 1 was 4.2% in 9- to 12-year old children, 7.0% in 1^st^ year students, and 2.8% in 20- to 55-year old adults. In arm 2, the prevalence was 7.8%, 9.9%, and 4.5%, respectively. In arm 3, the prevalence was 6.4%, 9.6%, and 4.5%, respectively. Additional baseline results of *S*. *haematobium* prevalence, infection intensity and microhaematuria, stratified by island and study group, are presented in Table 1 and S2 Table.

**Table 1:** Baseline characteristics.

Characteristic	Island	Stratification	9–12 year old children		1st year students		20–55 year old adults	
Schools/Shehias—no.*	Pemba and Unguja		88		88		89	
**Total participants—no.**	Pemba and Unguja		8278		6936		4015	
**Age—yr. (SD)**	Pemba and Unguja		10.5	(1.0)	7.7	(0.8)	34.1	(10.4)
**Sex—no.**	Pemba and Unguja	Women	4440		3598		2872	
** **		Men	3838		3338		1140	
**Participants with outcome data—no.**	Pemba and Unguja		8154		6813		3974	
*S*. *haematobium* infection**								
**Individuals infected—no./No. (%)**	Pemba and Unguja	MDA-only	120/2853	(4.2)	162/2325	(7.0)	38/1337	(2.8)
*** ***		Snail control	209/2688	(7.8)	226/2276	(9.9)	58/1330	(4.4)
*** ***		Behavior change	167/2613	(6.4)	213/2212	(9.6)	59/1307	(4.5)
	Pemba		328/4017	(8.2)	432/3543	(12.2)	102/1865	(5.5)
** **	Unguja		168/4137	(4.1)	169/3270	(5.2)	53/2109	(2.5)
**Arithmetic mean no. of eggs/10 ml of urine**	Pemba		8.2		13.2		1.0	
** **	Unguja		1.1		3.0		0.4	
Infection intensity—no./No (%)***	Pemba	Negative	3678/4004	(91.9)	3101/3533	(87.8)	1759/1861	(94.5)
** **		Light	219/4004	(5.5)	263/3533	(7.4)	95/1861	(5.1)
** **		Heavy	107/4004	(2.7)	169/3533	(4.8)	7/1861	(0.4)
** **	Unguja	Negative	3907/4069	(96.0)	3024/3190	(94.8)	2035/2088	(97.5)
** **		Light	143/4069	(3.5)	134/3190	(4.2)	49/2088	(2.3)
** **		Heavy	19/4069	(0.5)	32/3190	(1.0)	4/2088	(0.2)

* 2 schools and one shehia were not surveyed in Unguja at baseline.

** *S*. *haematobium*-positive is defined as urine filtration egg-positive or, in the absence of a urine filtration result, as hematuria-positive (trace, +, ++, +++).

*** The intensity of *S*. *haematobium* infection was categorized as negative (0 eggs per 10 ml of urine), light (1 to 49 eggs per 10 ml of urine), or heavy (≥50 eggs per 10 ml of urine) [22].

### Difference between treatment arms

**Fig 2:**
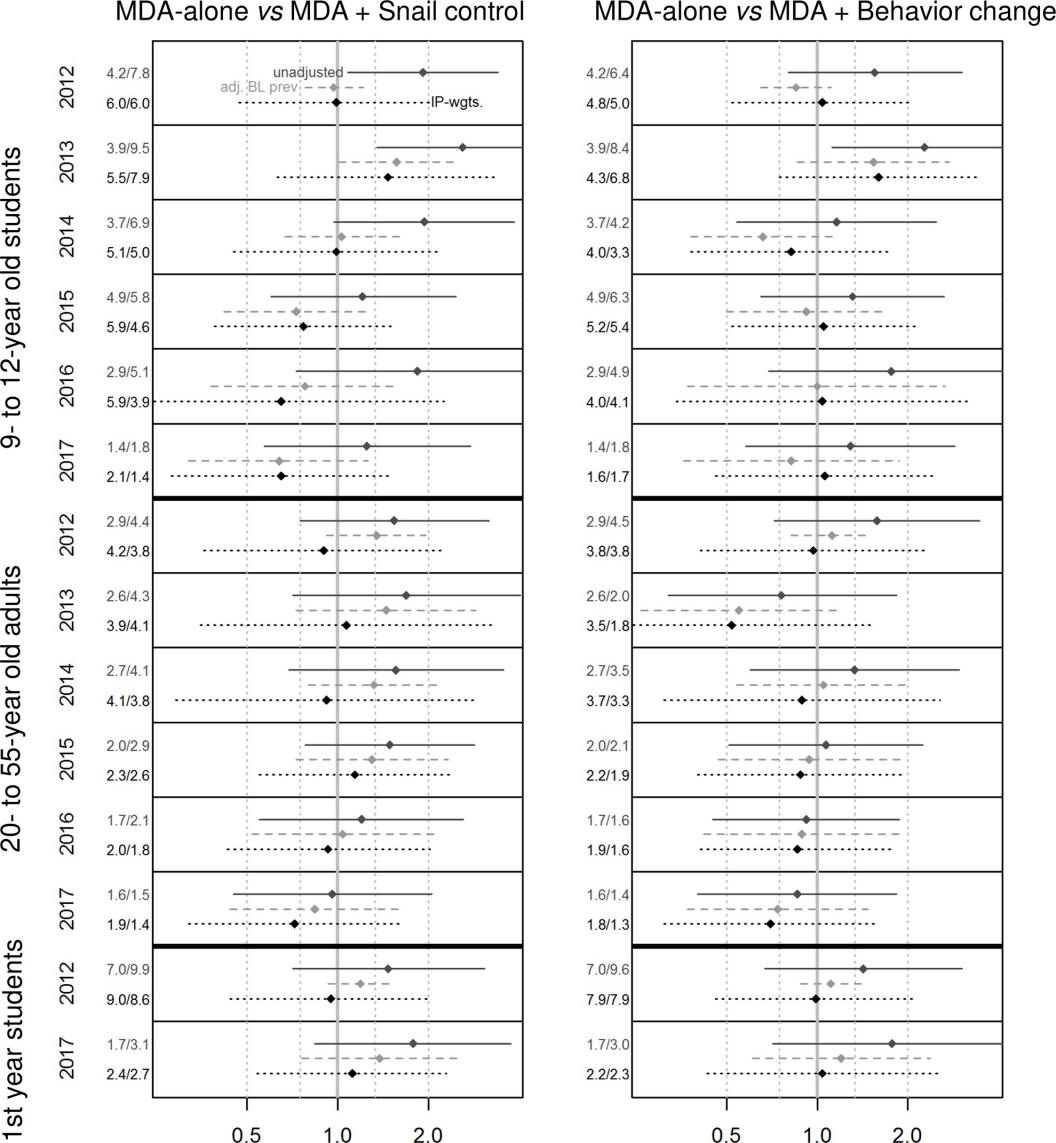
Odds ratios (OR) of *S*. *haematobium* infection. Data are unadjusted OR and weighted OR adjusted for baseline prevalence imbalance (inverse probability weights) and cluster size with 95% confidence intervals of the three participant groups in all study years. Treatment arms are biannual mass drug administration (MDA) plus snail control (arm 2) or biannual MDA plus behavior change (arm 3) *versus* biannual MDA-only (arm 1). Numbers represent the prevalence or the weighted prevalence, respectively, in a certain year (left: arm 1, right arm 2 or 3, respectively).

Fig 2 indicates that in none of the study years and in none of the study groups, there was a significant difference in the odds of *S*. *haematobium* infection, respectively, between the intervention arms. At the final survey of 9- to 12-year old children in 2017, the GEEs revealed no significant differences between the prevalence of arm 2 (OR 1.3, 95% CI 0.6–2.8) or arm 3 (OR 1.3, 95% CI 0.6–2.9) compared with arm 1 [14]. Also for 1^st^ year students no significant differences between the prevalence of arm 2 (OR 1.8, 95% CI 0.8–3.8) or arm 3 (OR 1.8, 95% CI 0.7–4.4) compared with arm 1 was detected in 2017. Similarly, for adults, there was no significant difference between the prevalence of arm 2 (OR 1.0, 95% CI 0.5–2.1) or arm 3 (OR 0.9, 95% CI 0.4–1.8) compared with arm 1. Adjusting for baseline prevalence and inverse probability weight, respectively, in 9- to 12-year old children and adults in 2017 and preceding years, did not reveal significant differences between the arms either. However, a trend of reduced odds of infection was observed for 9- to 12-year children attending schools in shehias with additional snail control and for adults living in shehias with additional behavioral interventions from 2015 till 2017 (S3 Table).

### Change in annual *S*. *haematobium* prevalence and intensity of infection

**Fig 3:**
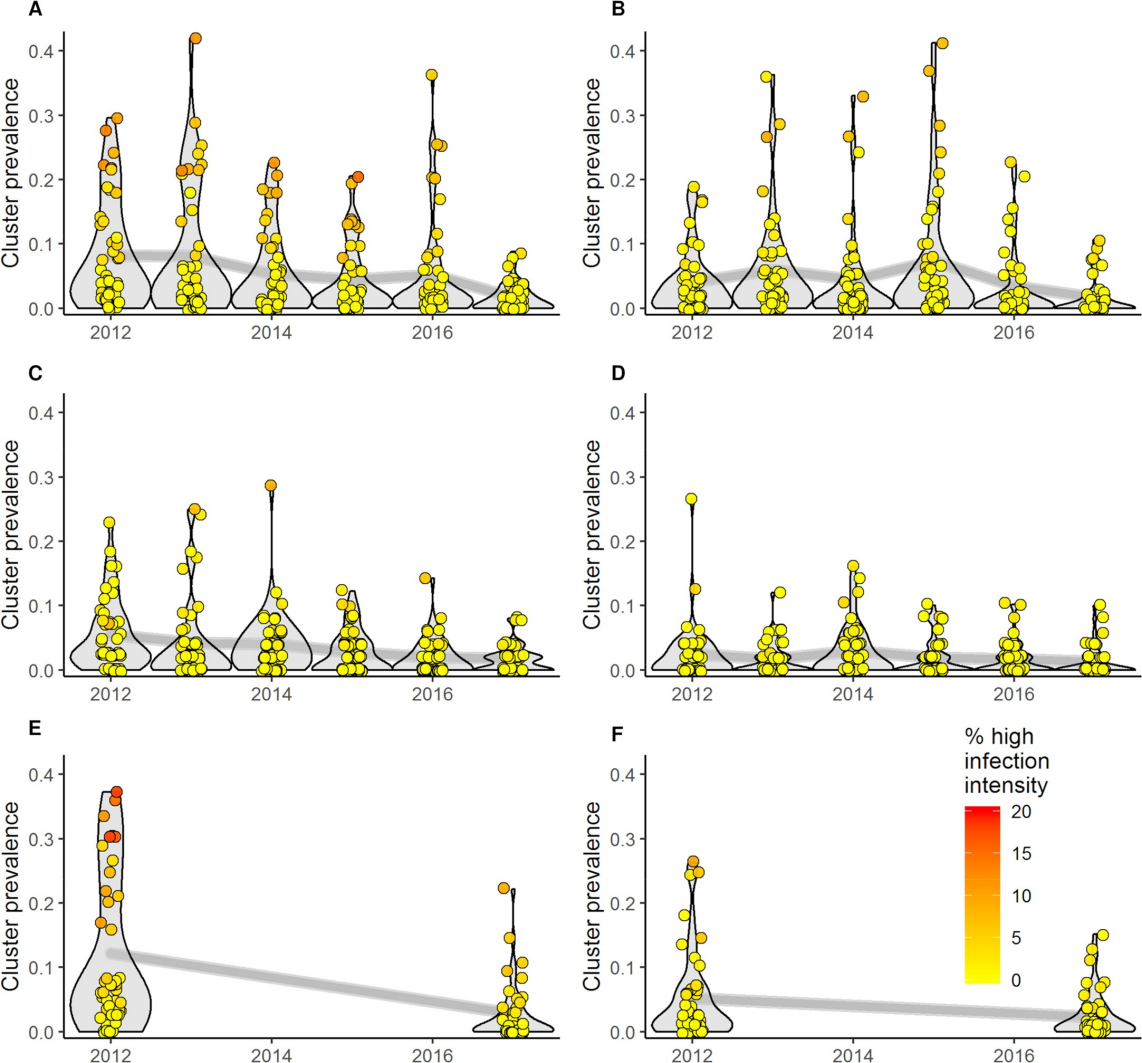
Change in *S*. *haematobium* prevalence and intensity. Data are prevalence and intensity in 45 schools/shehias (clusters) per island, stratified by population group, island, and year. The color code shows the percentage of heavily infected individuals among those infected. A) 9- to 12-year old students in Pemba; B) 9- to 12-year old students in Unguja; C) 20- to 55-year old adults in Pemba; D) 20- to 55-year old adults in Unguja; E) 1^st^ year students in Pemba; F) 1^st^ year students in Unguja.

Table 2 and the trend lines in Fig 3A–3F show that the overall *S*. *haematobium* prevalence decreased on Pemba and Unguja islands from 2011/12 to 2017 in all study populations. The relative difference from baseline to the final survey in Pemba was -79.5% (from 8.2% to 1.7%) in 9- to 12-year old children, -76.8% (from 12.2% to 2.8%) in 1^st^ year students, and -68.1% (from 5.5% to 1.7%) in adults. In Unguja the relative difference was -58.2% (from 4.1% to 1.7%) in 9- to 12-year old children, -55.5% (from 5.2% to 2.3%) in 1^st^ year students, and -48.7% (from 2.5% to 1.2%) in adults. The egg reduction rate from baseline to the final survey was 0.8%, 0.8%, and 0.5% for 9- to 12-year old children, 1^st^ year students, and adults in Pemba, and 0.4%, 07% and 0.4%, for respective groups, in Unguja. In 2017, heavy intensity infections occurred in 0.4% of 9- to 12-year old students, 1.2% of 1^st^ year students, and 0.2% of adults in Pemba, and in 0.3% of 9- to 12-year old children, 0.5% of 1^st^ year students, and 0.1% of adults in Unguja. Considering 9- to12-year old children, there were 5/45 schools in Pemba and 3/44 schools in Unguja where heavy infection intensities occurred in ≥1% of the students in 2017. Considering 1^st^ year students and adults, there were 13/45 schools and 4/45 shehias in Pemba and 7/44 schools and 2/45 shehias in Unguja, respectively, with heavy infection intensities ≥1%.

**Table 2:** Reduction of prevalence and intensity from baseline (2011/12) to final survey (2017).

**Pemba**	**9–12 year old**	** **	**1st year**	** **	**adults**	** **
Number tested at baseline*	4017		3543		1865	
Number infected at baseline*	328		432		102	
Prevalence at baseline* (95% CI)	8.2	(5.6–10.8)	12.2	(8.4–16.0)	5.5	(3.7–7.2)
Number tested in Year 6*	4888		4559		2237	
Number infected in Year 6*	82		129		39	
Prevalence in Year 6*(95% CI)	1.7	(1.0–2.3)	2.8	(1.4–4.3)	1.7	(1.1–2.4)
Absolute difference between prevalence at Year 6 and baseline*	-6.5		-9.4		-3.7	
Relative difference between prevalence in Year 6 and baseline (% change)*	-79.5		-76.8		-68.1	
Village level arithmetic mean infection intensity at baseline (including zeros)**	8.2		13.2		1.0	
Village level arithmetic mean infection intensity at Year 6 (including zeros)**	1.7		2.8		0.6	
Egg reduction rate (1- Year 6 intensity/baseline)**	0.8		0.8		0.5	
**Unguja**	**9–12 year old**	** **	**1st year**	** **	**adults**	
Number tested at baseline*	4137		3270		2109	
Number infected at baseline*	168		169		53	
Prevalence at baseline* (95% CI)	4.1	(2.5–5.6)	5.2	(2.9–7.5)	2.5	(1.1–3.9)
Number tested in Year 6*	4593		4171		2250	
Number infected in Year 6*	78		96		29	
Prevalence in Year 6*(95% CI)	1.7	(0.8–2.6)	2.3	(1.3–3.3)	1.3	(0.6–2.0)
Absolute difference between prevalence at Year 6 and baseline*	-2.4		-2.9		-1.2	
Relative difference between prevalence in Year 6 and baseline (% change)*	-58.2		-55.5		-48.7	
Village level arithmetic mean infection intensity at baseline (including zeros)**	1.1		3.0		0.4	
Village level arithmetic mean infection intensity at Year 6 (including zeros)**	0.7		0.9		0.2	
Egg reduction rate (1- Year 6 intensity/baseline)**	0.4		0.7		0.4	

95% CI: 95% confidence intervals

* *S*. *haematobium*-positive is defined as urine filtration egg-positive or, in the absence of a urine filtration result, as hematuria-positive (trace, +, ++, +++).

** The intensity of *S*. *haematobium* infection was categorized as negative (0 eggs per 10 ml of urine), light (1–49 eggs per 10 ml of urine), or heavy (≥50 eggs per 10 ml of urine) [22].

### Temporal and spatial heterogeneity

The violin plots in Fig 3A–3F indicate that the number of schools/shehias with high prevalence decreased over the years. Similarly, the number of clusters with a high percentage of heavy infection intensities was reduced. However, temporal heterogeneity was observed.

**Fig 4:**
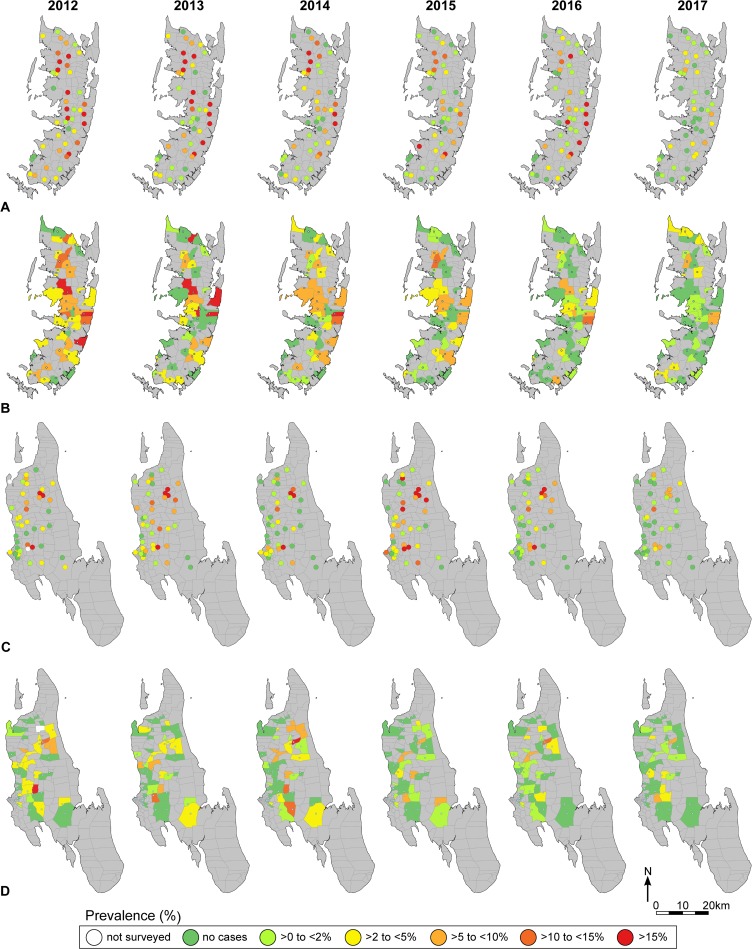
*S*. *haematobium* prevalence. Maps showing the annual prevalence in 45 schools/shehias in Pemba and Unguja, respectively, for each of the surveyed population groups. A) 9- to 12-year old children in Pemba; B) 20- to 55-year old adults in Pemba; C) 9- to 12-year old children in Unguja; D) 20- to 55-year old adults in Unguja. Maps were created with QGIS version 2.14.21 using coordinates of schools collected with a handheld Garmin GPSMAP 62sc device (Garmin, Kansas City, USA) and shape files of shehias provided by the Zanzibar Health Management Information System to the Neglected Diseases Program of the Zanzibar Ministry of Health.

The maps in Fig 4A–4C show that there was also spatial heterogeneity. The maps indicate the change in the *S*. *haematobium* prevalence in all 90 study schools/shehias stratified by study population over time. Most schools/shehias had a baseline prevalence of <10% and decreased to <5% in 2017; also some schools with a baseline prevalence >10% decreased to <5% in 2017. In 2017, considering 9- to 12-year old children, 37/44 schools in Unguja and 42/45 schools in Pemba had a prevalence of <5%. Considering 1^st^ year students in 2017, 37/44 schools in Unguja and 37/45 schools in Pemba had a prevalence of <5%. Considering adults in 2017, 42/45 shehias in Unguja and 42/45 shehias in Pemba had a prevalence of <5%. However, some schools/shehias remained at or bounced back to a prevalence ≥5% in certain years.

### Treatment coverage

**Fig 5:**
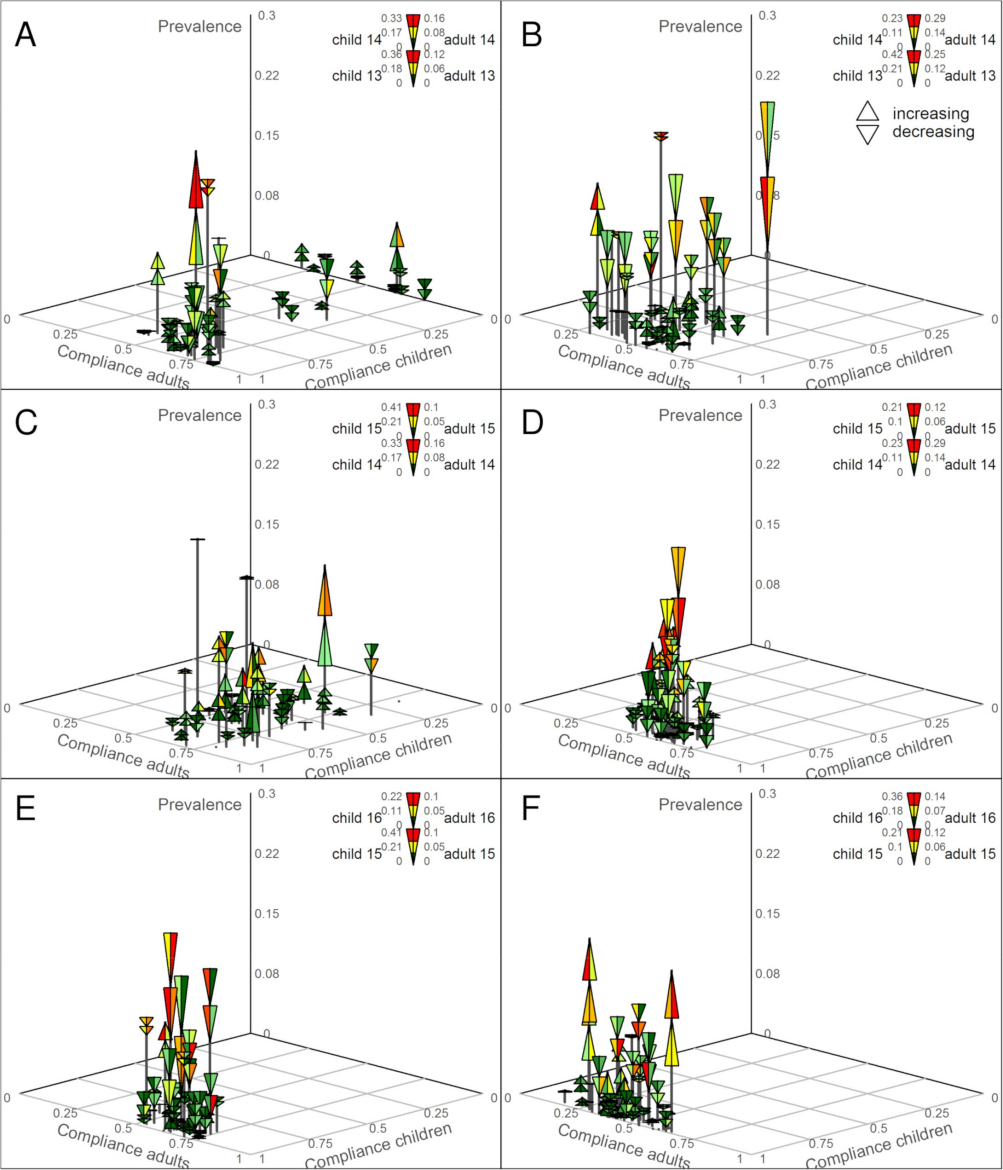
Prevalence *versus* compliance for 9- to12-year old children and for 20- to 55-year old adults from 2013 through 2015. The lower end of the triangle shows the higher prevalence of both years ((prevalence of children + prevalence of adults)/2); the upper end of the triangle shows the lower prevalence of both years. If triangles peak to the bottom, the prevalence decreased; if triangles peak to the top, prevalence increased. The color of the left bottom triangle shows the prevalence in schoolchildren in the year preceding the treatment (red = highest, dark green = lowest); the color of the right bottom triangle shows the prevalence in adults in the year preceding the treatment (red = highest, dark green = lowest); the color of the left top triangle shows the prevalence in schoolchildren in the year following the treatment (red = highest, dark green = lowest); the color of the right top triangle shows the prevalence in adults in the year following the treatment (red = highest, dark green = lowest). A: Coverage of directly observed school-based treatment (SBT) in 45 study schools and compliance with praziquantel treatment in community-wide treatment (CWT) in 45 study shehias in Unguja in November 2013 and the *S*. *haematobium* prevalence in 2013 and 2014. B: Coverage of directly observed SBT in 45 study schools and compliance with praziquantel treatment in CWT in 45 study shehias in Pemba in November 2013 and the *S*. *haematobium* prevalence in 2013 and 2014. C: Compliance with praziquantel treatment in CWT in 45 study shehias in Unguja in December 2014 and the *S*. *haematobium* prevalence in 2014 and 2015. D: Coverage of directly observed SBT in 45 study schools and compliance with praziquantel treatment at health posts in 45 study shehias in Pemba in December 2014 and the *S*. *haematobium* prevalence in 2014 and 2015. E: Coverage of directly observed SBT in 45 study schools and compliance with praziquantel treatment in CWT in 45 study shehias in Unguja in December 2015 and the *S*. *haematobium* prevalence in 2015 and 2016. F: Coverage of directly observed SBT in 45 study schools and compliance with praziquantel treatment in CWT in 45 study shehias in Pemba in December 2015 and the *S*. *haematobium* prevalence in 2015 and 2016.

Fig 5A, 5B and 5D–5F show that the praziquantel coverage of 9- to 12-year old children receiving directly observed SBT in the 45 study schools in Pemba and Unguja, respectively, improved over the years and was mostly >75% in 2015. The average coverage in Pemba was 85.8% in 2013, 93.9% in 2014, and 96.8% in 2015. In Unguja, some schools did not receive treatment in 2013; the average coverage was 61.9%. In 2014, only CWT but no SBT was conducted; the average coverage was 68.7%. In 2015, all schools were targeted and the average coverage was 92.7%.

Praziquantel compliance of 20- to 55-year old adults receiving CWT in 2013 and 2015 (Fig 5A, 5B, 5C, 5E and 5F) or praziquantel via health posts in Pemba in 2014 (Fig 5D) was <75% in most shehias in all years. The average compliance in Pemba was 48.8% in 2013, 58.8% in 2014, and 42.3% in 2015. In Unguja, the average compliance was 61.5%, 69.2%, and 62.3%, respectively.

Participants who did not receive treatment were at a considerably higher risk of harboring a *S*. *haematobium* infection compared with treated participants. The 9- to 12-year old children who participated at the SBT rounds in 2013 till 2015 preceding our annual survey had lower odds of infection (OR: 0.77; 95% 0.58–1.0). Adults who complied with praziquantel treatment had significantly lower odds of infection (OR: 0.68; 95% CI: 0.53–0.87).

## Discussion

The Zanzibar islands are one of the first settings in sub-Saharan Africa aiming for elimination of urogenital schistosomiasis as public health problem and interruption of transmission. To help achieve this goal the ZEST alliance was formed. From 2012 onward praziquantel was administered in two annual treatment rounds to the whole eligible population by the Neglected Diseases Program of the Zanzibar Ministry of Health. In addition, within a cluster randomized intervention trial conducted from 2011/12 through 2017, randomized shehias received snail control or behavior change interventions [10]. We assessed the impact of the interventions on the *S*. *haematobium* prevalence and intensity in schoolchildren and adults from 90 schools and shehias, respectively, from 2011/12 through 2017. Annually, more than 12,000 individuals were included in cross-sectional parasitological surveys.

Over this five year period, urogenital schistosomiasis was eliminated as public health problem from most of the study sites in Pemba and Unguja. The *S*. *haematobium* prevalence was significantly reduced, but transmission was not interrupted and infections persisted despite intense control interventions.

A trend of lower odds of *S*. *haematobium* infection in children visiting schools in shehias that received snail control in addition to biannual MDA in comparison with participants from biannual MDA-only shehias was observed from 2015 onward, when the GEES were adjusted for baseline prevalence. However, confidence intervals were wide and the difference between the arms was not statistically significant. Similarly, adults living in areas with behavioral interventions showed lower odds of infection from 2015 onward than their counterparts who only received biannual MDA, but the difference was not significant. Noteworthy, working in an elimination setting with a very low overall prevalence, it was clear from initial design that our study was not powered to detect the statistical significance of small differences between the study arms [10], and of course only small differences could be observed at these levels.

Our results show, that while transmission was not interrupted in the study period and at the scale the interventions were applied, island-wide biannual CWT and SBT, plus additional snail control or behavior change measures in randomized shehias, were able to reduce significantly the overall *S*. *haematobium* prevalence and infection intensity in all participant groups from baseline in 2011/12 till the final survey in 2017. On both, Pemba and Unguja, the overall prevalence was <3% in all participant groups in 2017.

Moreover, in most schools and shehias, urogenital schistosomiasis had been eliminated as a public health problem in 2017. The 1^st^ year students were identified as the group carrying most of the heavy infection intensities. Considering this participant group, 13/45 schools in Pemba and 7/44 schools in Unguja still had students with heavy infection intensities ≥1% in 2017. It seems that praziquantel distribution in CWT does not effectively reach children who are not (yet) attending primary school and receiving SBT. On Pemba and Unguja, an effort to increase coverage of preschool-aged children was made by including nursery and madrassa schools in the biannual treatment rounds since 2015. In future, the reach needs to be further increased so that young children benefit more from treatment. In addition, future island-wide or hotspot-focused behavioral and snail control interventions can help to educate children, parents, teachers and the general community about urogenital schistosomiasis transmission and prevention and consolidate treatment by lowering the risk of reinfection, respectively. While a study published in 2008 reported urogenital schistosomiasis to be rare in preschool-aged children [24] and we detected a low overall prevalence in all surveyed population groups in 2017, a new and larger study assessing the extent of *S*. *haematobium* infections throughout all age-groups is needed to provide greater insight into which sectors of the community infections are occurring.

A clear limitation of our study that might have resulted in some bias of our sample is that we recruited children in schools and adults in their homes. Non-school attenders and adults being out of their homes were not included, but might harbor heavy infections and contribute significantly to transmission [6]. Moreover, we used basic parasitological methods for diagnosis, which are not very sensitive, particularly for detecting ultra-light intensity infections [21]. Hence, the *S*. *haematobium* prevalence in our study is likely underestimated. Whether or not such undiagnosed ultra-light intensity infections significantly contribute to transmission is, however, unclear [25].

Importantly, our study showed that there was considerable temporal and also spatial heterogeneity. In some schools and shehias, the prevalence decreased in some years but bounced back to considerably high levels in other years. These shehias and schools were mostly not individually isolated, but clustered in areas covering neighbouring shehias in the north, center and east of Pemba and in the north and center of Unguja. Treatment coverage at cluster level did not directly explain an increase in prevalence. More likely to be responsible for the existence and persistence of hotspots are factors such as frequent human contact with natural open freshwater bodies, where intermediate host snails live and transmission occurs. The frequency of water contact is likely determined by the proximity of households and schools to water bodies, attractiveness of the water bodies for farming, household and leisure activities, and inconvenient access to safe water and alternative leisure options [26–28]. While these factors were not explored in our study, the presence of intermediate host snails at human water contact sites and their patent infections with *S*. *haematobium* were assessed in the snail control shehias [10]. We found that very few snails (<1%) were shedding cercariae and observed that snails repopulated treated sites soon after mollusciciding [14]. Hence, the risk of reinfection of humans and snails remains, and focal and repeated snail control should contribute to further reducing transmission in remaining hotspot areas.

In future, to achieve elimination as a public health problem across Pemba and Unguja, interventions will need to be improved and sustained. Biannual MDA alone, despite excellent coverage in schools and moderate coverage in adults over 5 years, was not always sufficient to achieve this goal. While in our elimination setting and CRT, the effect of snail control and behavioral interventions on prevalence and intensity was smaller than expected and statistically not significant, the interventions are likely to have a greater impact if applied at different scale and for a longer period. Recent reviews and a meta-analysis have shown that chemical mollusciciding is an effective way to control schistosomiasis and reduce prevalence [29, 30]. Behavior change and improving knowledge, attitudes and practices take time, but are essential since community engagement and compliance with interventions are key to sustained success. Combining all available interventions, including biannual MDA, snail control, behavioral change, and if possible, also improved access to safe water and sanitation, with best possible coverage and across neighboring hotspot shehias, might contribute to a further and sustainable reduction of prevalence and infection intensity.

In parallel to focusing improved intervention packages to hotspot areas, it will be important to start establishing surveillance-response as intervention in areas with very low prevalence. Moving towards interruption of transmission as the ultimate goal, a system will need to be in place that is able to diagnose and treat infected individuals rapidly, in order to avoid reintroduction and recrudescence in areas that become free of transmission [30–32]. To sustain and advance the gains made by ZEST, interventions must be continued long-term, improved, and adapted to the micro-epidemiology of *S*. *haematobium* in Zanzibar. Expansion of the MDA program to reach pre-school age children effectively is an essential requirement in Zanzibar and other endemic countries.

## Supporting information

S1 Table Table showing enrolment and exclusion of study participants. Study arms: 1 = Biannual mass drug administration (MDA)-only; 2 = biannual MDA plus snail control; 3 = biannual MDA plus behavior change interventions; UF: a single urine filtration of 10 ml urine.
(PDF)

S2 Table Table showing details of baseline characteristics. * 2 schools and one shehia were not surveyed in Unguja at baseline; ** *S*. *haematobium*-positive is defined as urine filtration egg-positive or, in the absence of a urine filtration result, as hematuria-positive (trace, +, ++, +++); *** The intensity of *S*. *haematobium* infection was categorized as negative (0 eggs per 10 ml of urine), light (1 to 49 eggs per 10 ml of urine), or heavy (≥50 eggs per 10 ml of urine).
(PDF)

S3 Table Table showing unadjusted, adjusted and inverse probability weights of *S. haematobium* infection in arm 2 and arm 3 compared with arm1. Adjusted odds ratios were calculated using the cluster prevalence at baseline as a continuous predictor in the model. For the inversed probability weighted odds ratios inverse probability weights were calculated for baseline prevalence and number of observations per cluster. The product of both weights was included in the model. All models used Generalized Estimating Equations with robust standard errors to account for clustering.
(XLSX)

S1 STROBE Checklist Checklist indicating, where items were included in the originally submitted Word file of the manuscript. (PDF)
