# Supplementary material for: A 5-Year intervention study on elimination of urogenital schistosomiasis in Zanzibar: Parasitological results of annual cross-sectional surveys
Source: PLoS Negl Trop Dis. 2019 May 6;13(5):e0007268. doi: 10.1371/journal.pntd.0007268 (PMC6502312; doi:10.1371/journal.pntd.0007268)
Supplement: S1 Table — Study arms: 1 = Biannual mass drug administration (MDA)-only; 2 = biannual MDA plus snail control; 3 = biannual MDA plus behavior change interventions; UF: a single urine filtration of 10 ml urine. (PDF) [file pntd.0007268.s001.pdf]

Table S1 Enrollment and exclusion of study participants

				Number of children aged 9-12 years					Number of 1st year children				Adults	Number of adults aged 20-55 years				
Island	Year	Study arm	Number of schools	Enrolled		Excluded			Enrolled		Excluded		Number of shehias	Enrolled		Excluded		
				Male	Female	No consent	Age not 9-12 years	No dipstick and UF	Male	Female	No consent	No dipstick and UF		Male	Female	Sex not known	Age not 20-55 years	No dipstick and UF
Pemba	2011/12	1	15	627	810	385	11	17	572	651	536	19	15	175	441	2	124	2
Pemba	2011/12	2	15	607	669	353	53	32	531	599	540	14	15	235	383	0	121	0
Pemba	2011/12	3	15	592	712	339	15	16	565	625	458	17	15	212	416	1	113	0
Unguja	2011/12	1	15	674	742	110	2	29	555	547	194	26	15	183	536	0	16	11
Unguja	2011/12	2	14	659	753	126	0	21	545	601	181	23	15	168	544	0	16	20
Unguja	2011/12	3	14	624	685	124	0	9	525	497	184	24	14	163	515	0	11	8
Pemba	2013	1	15	627	810	222	1	19					15	383	340	0	21	1
Pemba	2013	2	15	607	669	207	5	14					15	352	375	0	15	0
Pemba	2013	3	15	592	712	263	0	29					15	357	359	0	33	1
Unguja	2013	1	15	723	795	107	3	19					15	220	519	0	2	8
Unguja	2013	2	15	737	817	106	1	33					15	176	558	0	4	18
Unguja	2013	3	15	699	779	101	2	53					15	219	520	0	2	8
Pemba	2014	1	15	819	922	194	1	14					15	301	441	0	5	0
Pemba	2014	2	15	831	862	148	1	19					15	301	445	0	6	2
Pemba	2014	3	15	725	907	166	0	11					15	282	464	0	3	1
Unguja	2014	1	15	689	772	157	0	5					15	263	479	0	5	2
Unguja	2014	2	15	704	843	140	0	3					15	248	499	0	1	0
Unguja	2014	3	15	729	792	129	0	1					15	246	493	0	11	0
Pemba	2015	1	15	817	883	141	4	25					15	295	449	0	3	0
Pemba	2015	2	15	748	932	158	3	15					15	352	391	0	6	0
Pemba	2015	3	15	822	871	89	0	22					15	325	417	0	5	0
Unguja	2015	1	15	756	792	133	0	3					15	351	394	0	4	0
Unguja	2015	2	15	812	856	107	1	2					15	345	404	0	0	0
Unguja	2015	3	15	755	759	161	0	0					15	323	423	0	3	0
Pemba	2016	1	15	759	845	181	0	16					15	368	377	0	4	0
Pemba	2016	2	15	709	908	161	0	12					15	393	353	0	1	0
Pemba	2016	3	15	774	825	145	2	8					15	389	353	0	5	0
Unguja	2016	1	15	774	875	123	0	0					15	307	441	0	2	0
Unguja	2016	2	15	818	881	141	0	0					15	325	423	0	0	0
Unguja	2016	3	14	769	788	83	1	0					15	297	448	0	4	0
Pemba	2017	1	15	802	835	134	0	7	777	747	147	18	15	466	279	0	4	0
Pemba	2017	2	15	748	862	103	0	17	737	769	101	16	15	436	307	0	6	3
Pemba	2017	3	15	791	850	99	1	13	758	771	110	13	15	476	273	0	1	0
Unguja	2017	1	15	779	768	117	1	1	737	672	139	29	15	345	406	0	0	0
Unguja	2017	2	15	773	834	118	6	2	749	700	121	19	15	329	421	0	0	0
Unguja	2017	3	14	709	730	95	2	0	650	663	95	9	15	302	447	0	1	0

Study arms: 1 = Biannual mass drug administration (MDA)-only; 2= biannual MDA plus snail control; 3= biannual MDA plus behavior change interventions
UF: a single urine filtration of 10 ml urine
