# Supplementary material for: A 5-Year intervention study on elimination of urogenital schistosomiasis in Zanzibar: Parasitological results of annual cross-sectional surveys
Source: PLoS Negl Trop Dis. 2019 May 6;13(5):e0007268. doi: 10.1371/journal.pntd.0007268 (PMC6502312; doi:10.1371/journal.pntd.0007268)
Supplement: S2 Table — * 2 schools and one shehia were not surveyed in Unguja at baseline; ** S. haematobium-positive is defined as urine filtration egg-positive or, in the absence of a urine filtration result, as hematuria-positive (trace, +, ++, +++); *** The intensity of S. haematobium infection was categorized as negative (0 eggs per 10 ml of urine), light (1 to 49 eggs per 10 ml of urine), or heavy (≥50 eggs per 10 ml of urine). (PDF) [file pntd.0007268.s002.pdf]

Table S2. Detailed baseline characteristics

Characteristic			9- to 12-year old children		1st year students		20- to 55-year old adults	
**Schools/Shehias -- no.***	Pemba		45		45		45	
	Unguja		43		43		44	
**Total participants -- no.**	Pemba		4082		3593		1867	
	Unguja		4196		3343		2148	
**Age -- yr. (SD)**	Pemba		10.64	0.97	7.67	0.87	34.14	10.18
	Unguja		10.42	0.99	7.82	0.76	34.01	10.65
**Sex -- no.**	Pemba	Women	2236		1899		1242	
		Men	1846		1694		622	
	Unguja	Women	2204		1699		1630	
		Men	1992		1644		518	
**Participants with outcome data -- no.**	Pemba		4017		3543		1865	
	Unguja		4137		3270		2109	
S. haematobium* infection*								
**Individuals infected -- no./No. (%)**	MDA-only		120/2853	4.21	162/2325	6.97	38/1337	2.84
	Snail control		209/2688	7.78	226/2276	9.93	58/1330	4.36
	Behavior change		167/2613	6.39	213/2212	9.63	59/1307	4.51
	Pemba		328/4017	8.17	432/3543	12.19	102/1865	5.47
	Unguja		168/4137	4.06	169/3270	5.17	53/2109	2.51
**Arithmetic mean no. of eggs/10 ml of urine**	Pemba		8.16		13.2		1.02	
	Unguja		1.07		2.96		0.37	
**Infection intensity — no./No (%)*****	Pemba	Negative	3678/4004	91.86	3101/3533	87.77	1759/1861	94.52
		Light	219/4004	5.47	263/3533	7.44	95/1861	5.1
		Heavy	107/4004	2.67	169/3533	4.78	7/1861	0.38
	Unguja	Negative	3907/4069	96.02	3024/3190	94.8	2035/2088	97.46
		Light	143/4069	3.51	134/3190	4.2	49/2088	2.35
		Heavy	19/4069	0.47	32/3190	1	4/2088	0.19
**Microhaematuria intensity — no./No (%)**	Pemba	Negative	3572/4017	88.92	3014/3543	85.07	1598/1864	85.73
		Trace	134/4017	3.34	157/3543	4.43	84/1864	4.51
		(+)	105/4017	2.61	78/3543	2.2	86/1864	4.61
		(++)	110/4017	2.74	167/3543	4.71	67/1864	3.59
		(+++)	96/4017	2.39	127/3543	3.58	29/1864	1.56
	Unguja	Negative	3828/4129	92.71	3140/3256	96.44	1888/2108	89.56
		Trace	Nov 29	0.27	Mrz 56	0.09	49/2108	2.32
		(+)	93/4129	2.25	23/3256	0.71	52/2108	2.47
		(++)	92/4129	2.23	38/3256	1.17	52/2108	2.47
		(+++)	105/4129	2.54	52/3256	1.6	67/2108	3.18

* 2 schools and one shehia were not surveyed in Unguja at baseline
** *S. haematobium*-positive is defined as urine filtration egg-positive or, in the absence of a urine filtration result, as haematuria-positive (trace, +, ++, +++).
*** The intensity of *S. haematobium* infection was categorized as negative (0 eggs per 10 ml of urine), light (1 to 49 eggs per 10 ml of urine), or heavy (≥50 eggs per 10 ml of urine)
