# Supplementary material for: A 5-Year intervention study on elimination of urogenital schistosomiasis in Zanzibar: Parasitological results of annual cross-sectional surveys
Source: PLoS Negl Trop Dis. 2019 May 6;13(5):e0007268. doi: 10.1371/journal.pntd.0007268 (PMC6502312; doi:10.1371/journal.pntd.0007268)
Supplement: S1 STROBE Checklist — (PDF) [file pntd.0007268.s004.pdf]

STROBE Statement—Checklist of items that should be included in reports of ***cross-sectional studies***

	Item No	Recommendation
**Title and abstract**	1	(*a*) Indicate the study's design with a commonly used term in the title or the abstract<br><br>A 5-Year Intervention Study on Elimination of Urogenital Schistosomiasis in Zanzibar: Results of **Annual Cross-Sectional Surveys**<br><br>➔ Page 1
		(*b*) Provide in the abstract an informative and balanced summary of what was done and what was found<br><br>➔ Page 2
**Introduction**		
Background/rationale	2	Explain the scientific background and rationale for the investigation being reported<br>➔ Page 4 and 5
Objectives	3	State specific objectives, including any prespecified hypotheses<br>➔ Page 5
**Methods**		
Study design	4	Present key elements of study design early in the paper<br>➔ Page 6 and 7
Setting	5	Describe the setting, locations, and relevant dates, including periods of recruitment, exposure, follow-up, and data collection<br>➔ Setting: page 5/6<br>➔ Recruitment and follow-up: page 6-9
Participants	6	(*a*) Give the eligibility criteria, and the sources and methods of selection of participants<br>➔ Page 9 and published study protocol (http://www.biomedcentral.com/1471-2458/12/930)
Variables	7	Clearly define all outcomes, exposures, predictors, potential confounders, and effect modifiers. Give diagnostic criteria, if applicable.<br>➔ Page 10 and 11
Data sources/ measurement	8*	For each variable of interest, give sources of data and details of methods of assessment (measurement). Describe comparability of assessment methods if there is more than one group<br>➔ Page 11
Bias	9	Describe any efforts to address potential sources of bias<br>➔ Page 11
Study size	10	Explain how the study size was arrived at<br>➔ Published study protocol (http://www.biomedcentral.com/1471-2458/12/930)
Quantitative variables	11	Explain how quantitative variables were handled in the analyses. If applicable, describe which groupings were chosen and why<br>➔ Page 11
Statistical methods	12	(*a*) Describe all statistical methods, including those used to control for confounding<br>➔ Page 11
		(*b*) Describe any methods used to examine subgroups and interactions<br>➔ Page 11
		(*c*) Explain how missing data were addressed

		➔ Page 11
		(*d*) If applicable, describe analytical methods taking account of sampling strategy<br>➔ Page 11
		(*e*) Describe any sensitivity analyses<br>➔ Page 11
**Results**		
Participants	13*	(a) Report numbers of individuals at each stage of study—eg numbers potentially eligible, examined for eligibility, confirmed eligible, included in the study, completing follow-up, and analysed<br>➔ Table 1 and Table S1<br>➔ Table 2 and Table S2
		(b) Give reasons for non-participation at each stage<br>➔ Table S1
		(c) Consider use of a flow diagram<br>➔ Table S1 instead, too many numbers for flow diagram
Descriptive data	14*	(a) Give characteristics of study participants (eg demographic, clinical, social) and information on exposures and potential confounders<br>➔ Page 12, Table 1, Table 2, Table S2
		(b) Indicate number of participants with missing data for each variable of interest<br>➔ Table S1
Outcome data	15*	Report numbers of outcome events or summary measures<br>➔ Table 2 and Table S2<br>➔ Figures 1-4
Main results	16	(*a*) Give unadjusted estimates and, if applicable, confounder-adjusted estimates and their precision (eg, 95% confidence interval). Make clear which confounders were adjusted for and why they were included<br>➔ Table S3
		(*b*) Report category boundaries when continuous variables were categorized<br>➔ Page 11 and Table and Figure legends
		(*c*) If relevant, consider translating estimates of relative risk into absolute risk for a meaningful time period<br>➔ Not done
Other analyses	17	Report other analyses done—eg analyses of subgroups and interactions, and sensitivity analyses<br>➔ Table S3<br>➔ Figures 1-4
**Discussion**		
Key results	18	Summarise key results with reference to study objectives<br>➔ Page 20-24
Limitations	19	Discuss limitations of the study, taking into account sources of potential bias or imprecision. Discuss both direction and magnitude of any potential bias<br>➔ Page 20-24
Interpretation	20	Give a cautious overall interpretation of results considering objectives, limitations, multiplicity of analyses, results from similar studies, and other relevant evidence<br>➔ Page 20-24
Generalisability	21	Discuss the generalisability (external validity) of the study results<br>➔ Page 24

**Other information**		
Funding	22	Give the source of funding and the role of the funders for the present study and, if applicable, for the original study on which the present article is based<br>➔ See online submission form

*Give information separately for exposed and unexposed groups.

Note: An Explanation and Elaboration article discusses each checklist item and gives methodological background and published examples of transparent reporting. The STROBE checklist is best used in conjunction with this article (freely available on the Web sites of PLoS Medicine at http://www.plosmedicine.org/, Annals of Internal Medicine at http://www.annals.org/, and Epidemiology at http://www.epidem.com/). Information on the STROBE Initiative is available at www.strobe-statement.org.
